# Exploring the association between bioelectrical impedance parameters and body composition in women with and without dysmenorrhea and postmenopause

**DOI:** 10.14814/phy2.70473

**Published:** 2025-07-25

**Authors:** Suraiya Khatun, Akiko Uchizawa, Emi Kondo, Analiza M. Silva, Yuki Shimamura, Shogo Yamasaki, Yuki Ahagon, Daisuke Hoshi, Yoshiaki Tanaka, Naoki Mukai, Koichi Watanabe, Naomi Omi, Kiyoji Tanaka, Hiroyuki Sagayama

**Affiliations:** ^1^ Graduate School of Comprehensive Human Sciences University of Tsukuba Ibaraki Japan; ^2^ Institute of Health and Sport Sciences University of Tsukuba Ibaraki Japan; ^3^ Advanced Research Initiative for Human High Performance (ARIHHP) University of Tsukuba Tsukuba Japan; ^4^ Department of Sport Sciences Osaka University of Health and Sport Sciences Osaka Japan; ^5^ Exercise and Health Laboratory CIPER, Faculdade Motricidade Humana, Universidade de Lisboa Lisbon Portugal; ^6^ Department of Movement Sciences and Sports Training, School of Sport Sciences The University of Jordan Amman Jordan; ^7^ Japan Society for the Promotion of Science Tokyo Japan; ^8^ Department of Sports Sciences and Research Japan Institute of Sports Sciences (JISS) Tokyo Japan; ^9^ Department of Physical Education, Faculty of Physical Education Tokyo Women's College of Physical Education Tokyo Japan

**Keywords:** bioelectrical impedance, body composition, dysmenorrhea, phase angle, postmenopause, vector analysis

## Abstract

Female reproductive status influences body composition, including fat mass (FM), fat‐free mass (FFM), and bone health. We aimed to examine differences in body composition, bone health, and bioelectrical impedance analysis (BIA) parameters, including phase angle (PhA), among women with healthy menstruation, dysmenorrhea, and postmenopause. Forty‐five female participants were included and divided into control (healthy menstruation; *n* = 13), dysmenorrhea (*n* = 14), and postmenopausal (*n* = 18) groups. Body composition, bone mineral density (BMD), bone mineral content (BMC), and BIA parameters were measured using dual‐energy X‐ray absorptiometry and multifrequency BIA. The postmenopausal group had significantly lower BMD, BMC, and PhA. In the control group, PhA was positively correlated with body mass, body mass index, FM, FM index, FFM, and FFM index, whereas no correlations were observed in the other groups. Classic bioelectrical impedance vector analysis (BIVA) parameters were consistent with FFM and FFM index, while specific BIVA aligned with body fat. Graphical BIVA analysis showed signs of edema and decreased body cell mass in the postmenopausal group. These findings highlight important differences in body composition, bone health, and bioelectrical parameters across reproductive stages, with a focus on managing muscle loss and edema according to reproductive status.

## 1 INTRODUCTION

Female reproductive patterns significantly impact a woman's social, emotional, and overall quality of life, as evidenced by a range of symptoms such as dysmenorrhea, heavy menstrual bleeding, and menopause‐related changes (Munro et al., 2011). Heavy menstrual bleeding, abdominal and back pain, and other symptoms can decrease one's quality of life and impact activities of daily living for up to 2–3 days each month in over 70% of women with dysmenorrhea (Munro et al., 2011). Dysmenorrhea symptoms include abdominal and back pain, vomiting, headache, and diarrhea. Prostaglandins (PGs), particularly prostaglandin E_2_ (PGE2) and prostaglandin F_2α_ (PGF_2α_) released from the uterine lining, are the primary causes of dysmenorrhea (Petraglia et al., 2017). These compounds play a pivotal role in the contraction of the endometrium and myometrial walls, which leads to blood vessel damage and gastrointestinal disturbances, resulting in pain and other dysmenorrheic symptoms (Petraglia et al., 2017). According to Wu et al. (2022), underweight (BMI of <18.5 kg/m^2^) women are more likely to experience dysmenorrhea symptoms than those who are overweight (BMI of 25–29.9 kg/m^2^). Although mild or primary dysmenorrhea does not typically alter the fat mass (FM), fat‐free mass (FFM), bone mineral content (BMC), or bone mineral density (BMD) (Singh et al., 2015), the severity of dysmenorrhea symptoms can affect the body composition (Rizki Purwaningtyas et al., 2025). Given the increasing prevalence of slender Japanese women (Yasuda, 2023), especially among the younger population, addressing body composition concerning dysmenorrhea is crucial.

Menopause typically occurs between the ages of 45 and 60 years and is marked by the permanent termination of menstruation owing to decreased estrogen from decreased ovarian follicle function, influenced by aging (Zhang et al., 2022). This transition is characterized by hormonal shifts, including increased follicle‐stimulating and luteinizing hormones, along with decreased estrogen and progesterone (Burger et al., 2002). The transition to menopause significantly alters body composition. Shieh et al. (2023) noted that changes in body composition during menopause, particularly the increase in FM and decrease in FFM, have a direct impact on BMD and fracture risk. Given the significant hormonal and body composition changes that impact bone health during menopause, it is crucial to explore these alterations in Japanese postmenopausal women.

Various techniques are currently employed to assess body composition, including anthropometry, dual‐energy X‐ray absorptiometry (DXA), computed tomography (CT), and bioelectrical impedance analysis (BIA) (Kuriyan, 2018). Notably, multifrequency BIA has gained popularity for measuring body composition and total body water due to its noninvasive and rapid assessment capabilities (Marini et al., 2013). This method uses alternating current at a frequency of 1–1000 kHz to measure resistance (R) and reactance (Xc), collectively known as raw BIA parameters (Khatun et al., 2024). R represents the body's resistance to electrical current, higher values of R indicating lower water content and higher FM (Lukaski, 1996). Xc reflects the capacitive properties of the cell membranes and tissue interfaces, influenced by the cellular integrity and water distribution between the intra‐ and extracellular spaces (Lukaski, 1996). Piccoli introduced the classic bioelectrical impedance vector analysis (BIVA) to examine body hydration status and nutritional status (Piccoli et al., 1994), using R and Xc. BIVA employs impedance vector analysis using an RXc‐score graph (Piccoli et al., 1994). This graph uses reference values known as tolerance ellipses or compares different groups through confidence ellipses (Piccoli et al., 1994). BIVA graphically assesses body cell mass and hydration status, offering an alternative to predictive equations (Marini et al., 2013; Piccoli et al., 1994). R and Xc are standardized by individual's height (R/H ohm/m and Xc/H ohm/m) to remove conductor length effects (Piccoli et al., 1994). These standardized values are plotted on the RXc point graph or probability graph (Piccoli et al., 1994), indicating 50%, 75%, and 95% tolerance ellipses of the reference population. In classic BIVA, the primary axis of tolerance ellipses reflects total body water fluctuation (Piccoli et al., 1994); a shift toward the lower pole and upper pole represents oedema and dehydration, respectively. Although the classic BIVA method has demonstrated limited effectiveness in measuring body fat (Marini et al., 2012), greater efficacy was observed as a surrogate for monitoring the body cell mass and body water. Another approach, specific BIVA (Buffa et al., 2013), accurately measures body fat (Buffa et al., 2013; Marini et al., 2013) by standardizing R and Xc against height and cross‐sectional measurements (waist circumference, upper arm circumference, and calf circumference), resulting in specific resistance (Rsp) and specific reactance (Xcsp) (Buffa et al., 2013; Marini et al., 2013). In specific BIVA, the major axis represents the changes in body fat (Buffa et al., 2013); higher values of body fat are indicated in the upper pole of the RXc graph. Meanwhile, the minor axis represents variations in cellular health, muscle mass, and the intracellular/extracellular water (ICW/ECW) ratio (Piccoli et al., 1994), with leftward vector displacement indicating higher ICW/ECW ratios. R and Xc can be used to calculate the phase angle (PhA), which represents cellular health and integrity (Lukaski, 1996). PhA is a reliable indicator of the ECW/ICW ratio, with higher PhA values suggesting a greater ICW relative to ECW, indicating healthy cellular function (Khatun et al., 2024). PhA serves as a predictive tool for early detection of cellular damage and conditions such as dehydration or overhydration. Some studies have investigated the FM or FFM (Singh et al., 2015; Takata et al., 2023) and BMD or BMC (Shieh et al., 2023) in women with dysmenorrhea and postmenopausal women. However, no studies have examined differences in PhA values or their relationship with body composition, nor the use of BIVA in female groups categorized by reproductive status. Given this research gap, we hypothesize that different reproductive statuses, such as dysmenorrhea or post‐menopause, have significant associations with body composition, bone health, PhA values, and vector representation.

Therefore, in this study, we aimed to clarify: (1) The differences and association between body composition, bone health, and BIA parameters in healthy menstruation, dysmenorrhea, and postmenopausal groups; and (2) the analysis of agreement between body composition variables measured by DXA and BIVA; along with an explanation of the differences in the body water and muscle mass using classic BIVA among these female groups.

## 2 MATERIALS AND METHODS

Forty‐five female participants were included in this study and categorized based on their reproductive status. The reproductive status included normal menstruation with no pain or abnormalities, as the control group (*n* = 13, age: 30.6 ± 10.5 years); presence of abnormalities during menstruation, such as abdominal and back pain, headache, and vomiting as the dysmenorrhea group (*n* = 14, age: 35.2 ± 10.5 years); and menopausal status, that is, permanent cessation of menstruation, as the menopausal group (*n* = 18, age: 60.9 ± 5.5 years). Women who had no history of dysmenorrhea symptoms and no irregular menstruation in the past 12 months were selected as control participants. Women with dysmenorrhea were selected based on reporting at least two dysmenorrhea symptoms during menstruation on the questionnaire and having a regular menstrual cycle. Menopausal women were chosen if they had been in menopause for at least 3 years. All participants were physically healthy, disease‐free, and not taking any medication, including oral contraceptives, or undergoing hormonal or menopausal replacement therapy. We excluded individuals with conditions such as uterine fibroids, menstrual dysfunction, amenorrhea, dwarfism, irregular menstruation, surgery, post‐childbirth menstruation cessation, diabetes, lung disease, liver disease, digestive disease, hyperlipidemia, and nerve and muscle diseases.

To ensure consistency in the measurement of body composition, BMC or BMD, R, and Xc values using the DXA and BIA devices, all the measurements were conducted in the morning with the participants in a fasting state (at least 10 h fasting). Participants were allowed to drink water up to 1 h before the measurement to ensure their hydration status. Participants were instructed to arrive at the institution in loose‐fitting attire, specifically a plain‐colored T‐shirt without any prints or embellishments and shorts free of metal elements such as zippers or buttons.

All participants signed a written informed consent form, and the protocol was approved by the Institutional Review Board of the Institute of Health and Sports Sciences of the University of Tsukuba (Ref No Tai 022–109).

### 2.1 Anthropometric measurements

The following anthropometric parameters were measured in this study: (a) height was measured using a stadiometer (DST‐210S, Muratec‐KDS Corp., Kyoto, Japan); (b) body mass was measured using a digital scale (MC‐780A‐N, TANITA‐Tokyo, Japan); and (c) waist, arm, and calf circumferences were measured using non‐stretchable measuring tape (SP‐715; Sekisui Jushi Corp., Osaka, Japan). The intraindividual technical error of measurement (TEM) and coefficient of variation (CV%) were calculated to assess the accuracy of two consecutive measurements: waist circumference, TEM = 0.2 and CV% = 0.32; arm circumference, TEM = 0.2 and CV% = 0.86; calf circumference, TEM = 0.2 and CV% = 0.45. Body mass index (BMI) was calculated by dividing body mass by height squared (kg/m^2^).

### 2.2 DXA

Body composition, BMD, and BMC were measured using DXA (Horizon; Hologic, Waltham, MA, USA). All the DXA measurements were performed by a certified radiation technician. DXA measurements included assessments of body composition such as fat%, FM, and FFM, as well as BMD and BMC measurements. These measurements covered various regions, including the whole‐body, arm, leg, rib, total spine, lumbar spine, and pelvis.

### 2.3 BIA

A multifrequency (5 kHz, 50 kHz, and 250 kHz) segmental body composition analyzer (MC‐780A‐N, TANITA Co., Ltd., Tokyo, Japan) was used to measure the R and Xc value. For analysis, we used R and Xc values at a frequency of 50 kHz. This BIA device demonstrated the accuracy of FFM variables with a concordance correlation coefficient of 0.922, precision of 0.962, and accuracy of 0.959 when compared with DXA (Khatun et al., 2024) (*n* = 19 young men). The device demonstrated, as the intraclass correlation coefficient (ICC) and CV for FFM were 0.993 and 0.50, respectively, on two consecutive days (*n* = 13, young men) in our previous study (Khatun et al., 2024).

PhA was calculated as: [arc tangent (Xc/R) × 180/π] and impedance vector (Z) was calculated as Z^2^ = R^2^ + Xc^2^ (Marini et al., 2013). In the classic BIVA (Piccoli et al., 1994), the R and Xc values were standardized based on the individual's height (R/H and Xc/H) to eliminate the effect of the conductor length. Additionally, in a specific BIVA (Marini et al., 2013), the R and Xc values were multiplied by a correction factor (A/L), where A represents the body area or A = 0.45 arm area + 0.10 waist area + 0.45 calf area, and L represents the conductor length or L = 1.1 H (body height in meters). The waist, arm, and calf areas were measured using the formula C^2^/4π (C is the circumference of the body segments).

The mean impedance vectors represented by the average values of R/H and Xc/H were plotted on a 95% confidence ellipse (RXc mean graph) for the three groups of women. Additionally, the 95%, 75%, and 50% tolerance ellipses for the control group (reference vector) were plotted to assess the probability of individual vectors of the dysmenorrhea and menopausal female participants falling within these limits (RXc point graph). The major axis of the vectors indicates the hydration status (Piccoli et al., 1994) (overhydrated individuals tend to deviate toward the lower pole, and vice versa). Additionally, the minor axis of the vector indicates the muscle mass (Marini et al., 2013).

### 2.4 Statistical analysis

Descriptive statistical analyses were conducted for all the participants' characteristics. The normality of the data was evaluated using the Kolmogorov–Smirnov and Shapiro–Wilk tests, along with Q‐Q plots. Differences in body composition, BMD, BMC, and BIA parameters (PhA, R, Xc, and Z) were examined using one‐way analysis of variance (ANOVA) for normally distributed data and the nonparametric Kruskal–Wallis test for data that were not normally distributed. Scheffe's test was performed as a post hoc test. The relationships between body composition and bone health were analyzed using Pearson's or partial correlations (adjusted for age; during analysis of all three groups, *n* = 45). Similarly, the correlation between classic and specific BIVA parameters with DXA body composition variables was assessed using Pearson's or partial correlation analysis. Additionally, zonoid depth–depth analysis was used to measure the agreement between BIVA and DXA parameters. Specific bioelectrical variables (Rsp, Xcsp, and Zsp) were computed using the specific BIVA software 1.0 (http://www.specificbiva.com), while classic bioelectrical variables (R/H, Xc/H, and Z/H) were calculated using BIVA software 2002 by Antonio Piccoli. Data analysis was performed using IBM SPSS version 29. Zonoid depth analysis was conducted using R Studio version 2025.05.0 + 496 (2025.05.0 + 496). The DepthProc, dplyr, ggplot2, and mass packages were used for analysis. Statistical significance was defined as *p* < 0.05.

## 3 RESULTS

The characteristics of the female participants are summarized as mean ± standard deviation (Table 1). No significant differences were observed in the characteristics of the control and dysmenorrhea groups. The postmenopausal group exhibited significantly lower BMD, BMC, and PhA values compared to the control and dysmenorrhea groups. The relationship between the body composition, BMD, and BMC parameters (Table 2) varied across the groups. In the control group, several BMD/BMC parameters positively correlated with body weight, BMI, fat mass index (FMI), FFM, and FFM index (FFMI). In contrast, the dysmenorrhea group exhibited positive correlations with the BMD/BMC parameters only in relation to the FFM and FFMI. The postmenopausal group demonstrated a negative correlation between fat%, BMD, and BMC at multiple sites: total spine BMD (*r* = −0.549), lumbar spine BMC (*r* = −0.500), and pelvic BMC (*r* = −0.538) (*p* < 0.05). However, this group showed a positive correlation with FFM and FFMI.

**TABLE 1:** Characteristics and comparison of the variables among the three groups.

Variables	All females (*n* = 45)	Control (*n* = 13)	Dysmenorrhea (*n* = 14)	Menopause (*n* = 18)	*p*
Anthropometric parameters					
Age, years	44.2 ± 16.4	30.6 ± 10.5^a^	35.2 ± 10.5^a^	60.9 ± 5.5^b^	**<0.001**
Height, cm	158.4 ± 5.9	157.4 ± 5.6	160.3 ± 5.3	157.6 ± 6.4	0.340
Body mass, kg	53.1 ± 7.1	52.8 ± 6.5	55.9 ± 8.3	51.2 ± 6.1	0.174
BMI, kg/m^2^	21.2 ± 2.7	21.3 ± 2.7	21.8 ± 3.2	20.6 ± 2.4	0.495
Waist circumference, cm	75.2 ± 8.4	72.9 ± 6.3	77.1 ± 10.8	75.5 ± 7.7	0.441
Leg circumference, cm	34.3 ± 3.1	33.8 ± 3	34.8 ± 3.4	34.3 ± 2.7	0.713
Arm circumference, cm	24.2 ± 2.4	24.5 ± 2.7	24.5 ± 2.6	23.9 ± 2.1	0.720
BIA parameters					
R_50_, Ω	697.4 ± 81.3	721.9 ± 74.3	717 ± 93	664.4 ± 68.2	0.081
Xc_50_, Ω	60.7 ± 9.4	67.8 ± 5.3^a^	64.3 ± 9.3^a^	52.7 ± 5^b^	**<0.001**
Z_50_, Ω	700.0 ± 81.5	725.1 ± 74.2	719.9 ± 93.1	666.5 ± 68.3	0.074
R/H, Ω/m	440.3 ± 48.6	458.8 ± 45.5	447.4 ± 56.8	421.4 ± 38.8	0.084
Xc/H, Ω/m	38.3 ± 5.8	43.1 ± 3.4^a^	40.1 ± 5.7^a^	33.5 ± 3^b^	**<0.001**
Z/H, Ω/m	442 ± 48.7	460.9 ± 45.5	449.3 ± 56.8	422.8 ± 38.9	0.077
Rsp, Ω. cm	432.7 ± 56.9	436.3 ± 38.2	454.7 ± 69.6	413.1 ± 53.3	0.155*
Xsp, Ω. cm	37.8 ± 7.4	41.4 ± 6.4^a^	40.9 ± 7.9^a^	32.9 ± 4.6^b^	**<0.001** *
Zsp, Ω. cm	434.4 ± 57.2	438.3 ± 38.6	456.6 ± 69.9	414.4 ± 53.4	0.149*
Phase angle, °	5.0 ± 0.6	5.5 ± 0.5^a^	5.1 ± 0.6^a^	4.5 ± 0.3^b^	**<0.001**
DXA parameters					
Fat, %	29.9 ± 5.1	28.8 ± 3.9	31.5 ± 5.4	29.6 ± 2.0	0.345
FM, kg	16.1 ± 4.3	15.3 ± 3.3	17.8 ± 5.1	15.3 ± 3.9	0.193
FMI, kg/m^2^	6.4 ± 1.7	6.2 ± 1.4	7.0 ± 2.1	6.2 ± 1.6	0.399
FFM, kg	37.0 ± 4.1	37.4 ± 3.9	38.1 ± 4.8	35.9 ± 3.7	0.297
FFMI, kg/m^2^	14.7 ± 1.4	15.1 ± 1.4	14.8 ± 1.6	14.4 ± 1.2	0.386
Whole body BMC, g	1834.9 ± 292.4	1981.7 ± 263.3^a^	1966.9 ± 220.4^a^	1626.4 ± 241.7^b^	**<0.001**
Whole body BMD, g/cm^2^	1.001 ± 0.097	1.068 ± 0.081^a^	1.029 ± 0.070^a^	0.928 ± 0.080^b^	**<0.001**
Arm BMC, g	111.8 ± 19.2	116.9 ± 17.3^a^	119.7 ± 15^a^	100 ± 18.6^b^	**0.004**
Arm BMD, g/cm^2^	0.642 ± 0.059	0.675 ± 0.045^a^	0.670 ± 0.036^a^	0.598 ± 0.056^b^	**<0.001**
Rib BMC, g	68.6 ± 14.1	76.4 ± 12.7^a^	75.5 ± 10.4^a^	57.5 ± 10.1^b^	**<0.001** *
Rib BMD, g/cm^2^	0.568 ± 0.070	0.601 ± 0.062^a^	0.591 ± 0.059^a^	0.525 ± 0.065^b^	**0.002**
Total spine BMC, g	96.1 ± 18.5	105.9 ± 18.1^a^	101.9 ± 18.4^a^	84.6 ± 12.3^b^	**0.001**
Total spine BMD, g/cm^2^	0.763 ± 0.104	0.822 ± 0.096^a^	0.794 ± 0.087^a^	0.697 ± 0.088^b^	**<0.001**
Lumbar spine BMC, g	49.3 ± 9.8	53.5 ± 11.5^a^	51.1 ± 6.9^a^	44.9 ± 8.9^b^	**0.033**
Lumbar spine BMD, g/cm^2^	0.978 ± 0.160	1.053 ± 0.175^a^	1.029 ± 0.968^a^	0.886 ± 0.148^b^	**0.004**
Pelvic BMC, g	197.2 ± 52.6	222.8 ± 52^a^	219.7 ± 44.9^a^	161.2 ± 37.7^b^	**<0.001** *
Pelvic BMD, g/cm^2^	1.077 ± 0.147	1.160 ± 0.157^a^	1.114 ± 0.131^a^	0.989 ± 0.102^b^	**0.002**
Leg BMC, g	321.1 ± 57.1	334.6 ± 56^a^	349.2 ± 47.2^a^	289.3 ± 51.7^b^	**0.006**
Leg BMD, g/cm^2^	1.007 ± 0.096	1.054 ± 0.089^a^	1.040 ± 0.073^a^	0.949 ± 0.089^b^	**0.002**
Year of menopause, years	‐	‐	‐	8.47 ± 7.89	

*Note*: The characteristic variables of the three groups of women are reported as mean ± SD. *p* values show the results of the differences between groups on analysis of variance or Kruskal–Wallis test. Mean values within a row with unlike superscript letters were significantly different between groups, independently, using post hoc Scheffe test (*p* < 0·05).

Abbreviations: BMC, bone mineral concentration; BMD, bone mineral density; FFM, fat‐free mass; FFMI, fat‐free mass index; FM, fat mass; FMI, fat mass index; R/H and Xc/H, Classic BIVA; R, Resistance; Rsp and Xcsp, specific BIVA; Xc, capacitance; Z, impedance vector.

Bold values indicate statistically significant.

* *p*: Nonparametric test.

**TABLE 2:** Relationship between body composition and bone health.

	Whole body		Arm		Rib		Total spine		Lumbar spine		Pelvic		Leg	
	BMC	BMD	BMC	BMD	BMC	BMD	BMC	BMD	BMC	BMD	BMC	BMD	BMC	BMD
All (*n* = 45)														
Body mass	**0.494** *	0.212	**0.562** *	0.242	**0.463** *	0.24	**0.379** *	0.117	0.258	0.204	**0.383** *	**0.373** *	**0.605** *	**0.482** *
BMI	**0.305** *	0.218	**0.350** *	0.195	**0.384** *	0.231	0.193	0.083	0.052	0.214	0.214	**0.320** *	0.297	0.289
Fat%	−0.025	−0.077	0.096	−0.054	0.137	−0.091	0.025	−0.191	−0.172	0.014	−0.167	0.219	−0.001	0.001
FM	0.201	0.035	**0.318** *	0.073	0.269	0.030	0.175	−0.08	−0.004	0.080	0.069	0.291	0.275	0.215
FMI	0.101	0.032	0.198	0.041	0.220	0.022	0.078	−0.096	−0.100	0.084	−0.017	0.248	0.115	0.111
FFM	**0.642** *	**0.330**	**0.635** *	**0.341** *	**0.515** *	**0.383**	**0.470** *	0.287	**0.452** *	0.267	**0.591** *	**0.337** *	**0.757** *	**0.606** *
FFMI	**0.477** *	**0.393** *	**0.441** *	**0.335** *	**0.481** *	**0.431** *	**0.283** *	0.288	0.232	**0.317** *	**0.446** *	**0.318** *	**0.442** *	**0.433** *
Control (*n* = 13)														
Body mass	**0.722** *	0.468	**0.829** *	0.432	**0.646** *	**0.568** *	**0.667** *	0.449	0.528	**0.598** *	**0.668** *	**0.660** *	**0.746** *	**0.620** *
BMI	0.542	0.535	**0.735** *	**0.587** *	**0.569** *	**0.616** *	0.542	0.481	0.312	**0.634** *	0.419	**0.591** *	0.394	0.381
Fat%	0.494	0.516	**0.664** *	0.505	0.524	**0.639** *	**0.575** *	**0.568** *	0.413	**0.618** *	0.395	**0.639** *	0.248	0.236
FM	**0.646** *	0.526	**0.826** *	0.513	**0.614** *	**0.636** *	**0.672** *	0.542	0.486	**0.640** *	0.550	**0.696** *	0.523	0.446
FMI	0.516	0.536	**0.727** *	**0.570** *	0.539	**0.630** *	**0.572** *	0.531	0.345	**0.628** *	0.388	**0.623** *	0.307	0.297
FFM	**0.659** *	0.335	**0.685** *	0.285	**0.559** *	0.408	0.544	0.29	0.471	0.456	**0.650** *	0.512	**0.805** *	**0.659** *
FFMI	0.504	0.471	**0.657** *	0.536	0.532	0.530	0.447	0.372	0.242	**0.565** *	0.400	0.489	0.436	0.422
Dysmenorrhea (*n* = 14)														
Body mass	0.491	0.265	**0.536** *	0.272	0.371	0.228	0.198	0.125	0.328	0.064	0.343	0.185	0.500	0.352
BMI	0.308	0.213	0.346	0.139	0.141	0.208	−0.055	0.015	0.123	0.073	0.254	0.173	0.321	0.265
Fat%	−0.271	−0.271	−0.01	−0.176	−0.264	−0.255	−0.216	−0.358	−0.377	−0.169	−0.282	0.061	−0.253	−0.216
FM	0.039	−0.077	0.223	−0.019	−0.026	−0.099	−0.062	−0.191	−0.088	−0.1	−0.02	0.094	0.063	0.009
FMI	−0.043	−0.095	0.121	−0.076	−0.132	−0.101	−0.179	−0.23	−0.172	−0.08	−0.056	0.077	−0.014	−0.024
FFM	**0.800** *	**0.537** *	**0.682** *	0.487	**0.664** *	0.497	0.406	0.417	**0.657** *	0.217	**0.610** *	0.218	**0.790** *	**0.594** *
FFMI	**0.691** *	**0.566** *	**0.547** *	0.389	0.468	**0.563** *	0.126	0.341	0.484	0.258	**0.598** *	0.252	**0.680** *	**0.577** *
Postmenopausal (*n* = 18)														
Body mass	0.378	0.078	0.451	0.138	0.452	0.052	0.460	−0.104	0.053	0.021	0.192	0.464	**0.595** *	**0.584** *
BMI	0.178	0.078	0.165	0.057	**0.504** *	0.057	0.173	−0.169	−0.182	−0.019	−0.016	0.295	0.272	0.331
Fat%	−0.254	−0.307	−0.229	−0.344	0.121	−0.453	−0.119	**−0.549** *	**−0.500** *	−0.305	**−0.538** *	0.106	−0.092	−0.019
FM	−0.004	−0.176	0.046	−0.177	0.271	−0.281	0.132	−0.411	−0.322	−0.21	−0.269	0.289	0.209	0.256
FMI	−0.100	−0.172	−0.094	−0.214	0.279	−0.267	−0.01	−0.424	−0.408	−0.213	−0.356	0.189	0.039	0.113
FFM	**0.629** *	0.317	**0.697** *	0.416	0.459	0.386	**0.620** *	0.264	0.430	0.258	0.603	0.460	0.762	0.693
FFMI	**0.494** *	0.395	0.461	0.411	**0.619** *	**0.486** *	0.359	0.252	0.205	0.258	0.463	0.327	0.489	0.503

*Note*: Values are shown as correlation coefficients. Pearson's or partial correlation coefficients after adjusting for age were conducted.

Abbreviations: BMC, bone mineral concentration (g); BMD, bone mineral density (g/cm^2^); FFM, fat‐free mass; FFMI, fat‐free mass index; FM, fat mass; FMI, fat mass index; LS, lumbar spine; TS, total spine; WB, whole body.

Bold values indicate statistically significant.

* *p* < 0.05.

**FIGURE 1:**
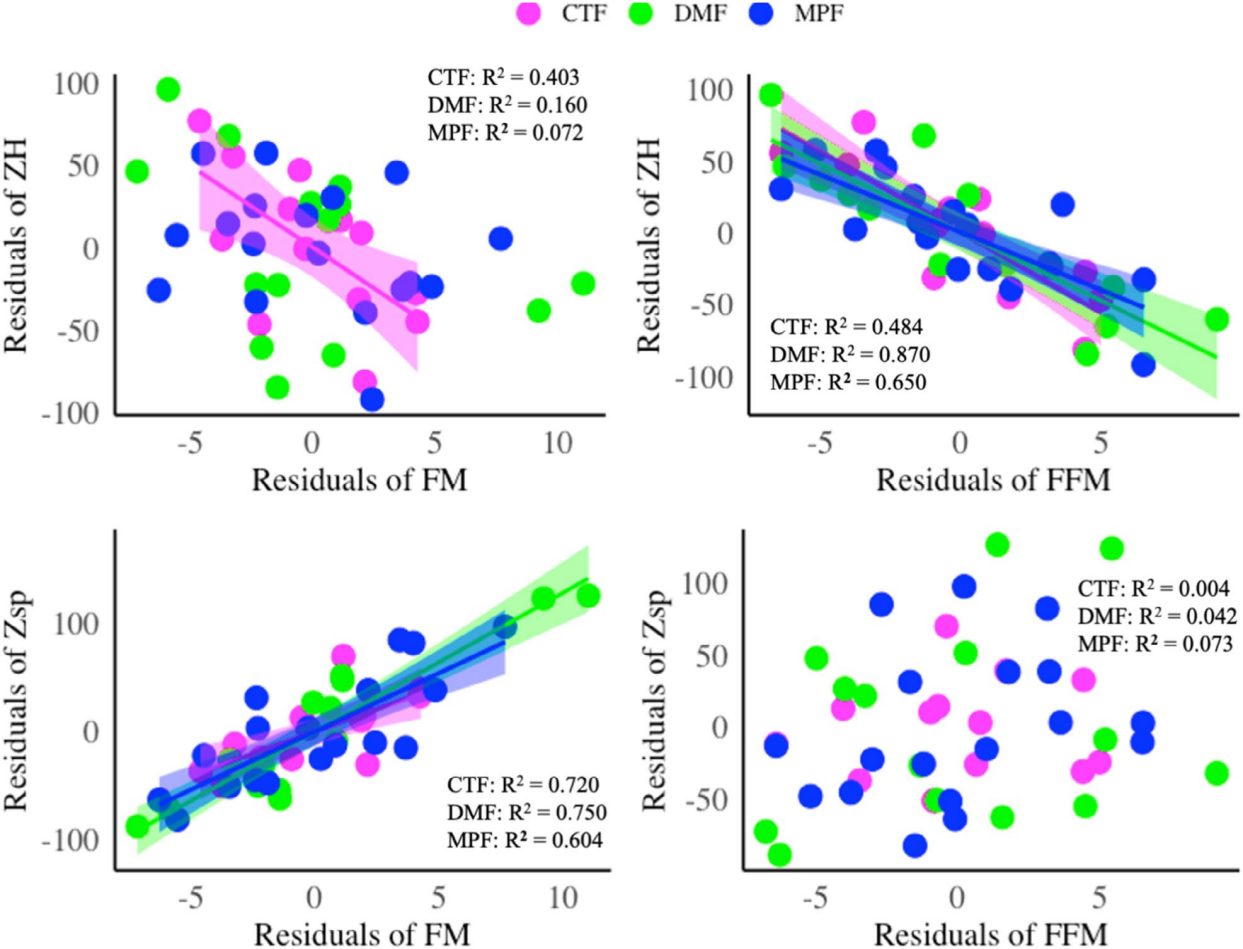
Zonoid depth analysis for measuring the agreement between classic (Z/H) and specific (Zsp) impedance vector with fat mass (FM) and fat‐free mass (FFM) measured by DXA. CTF: Magenta, DMF: Green, MPF: Blue, CTF: women without dysmenorrhea, DMF: women with dysmenorrhea, MPF: postmenopausal women.

**FIGURE 2:**
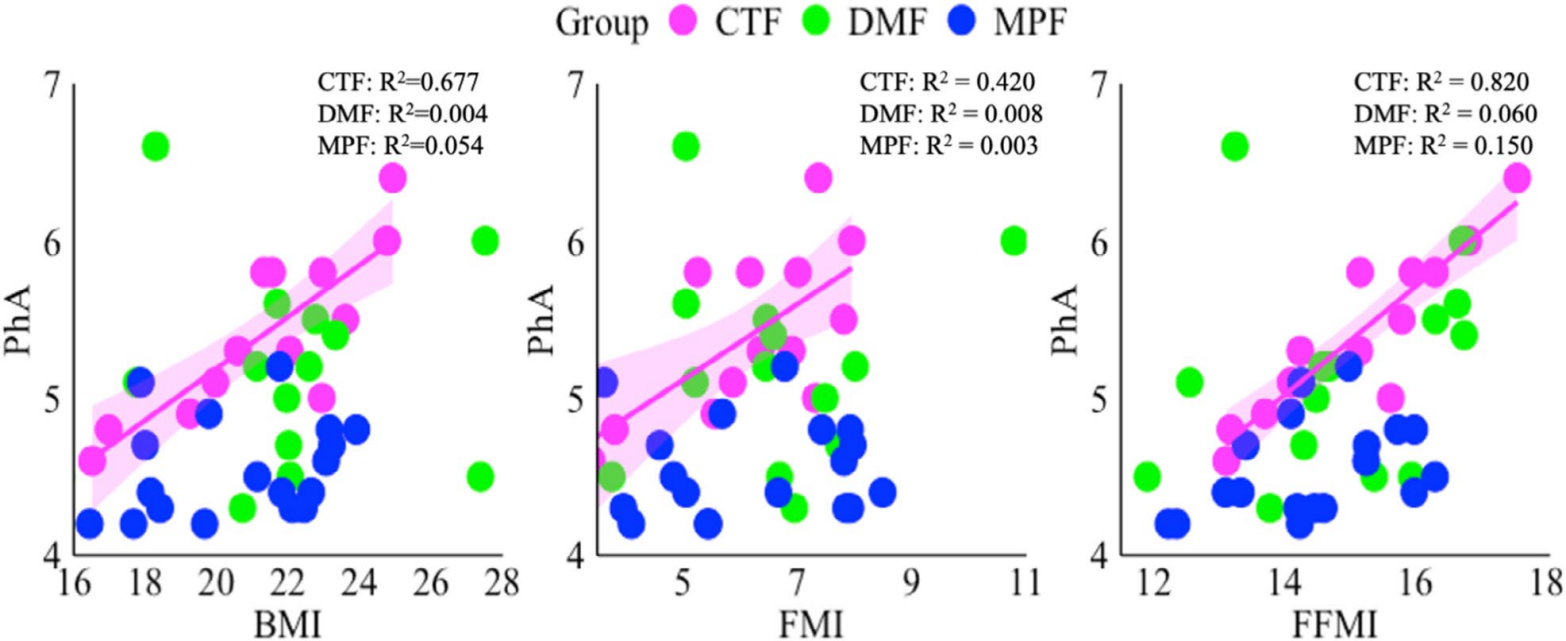
Correlation between body mass index (BMI), fat mass index (FFMI), and fat‐free mass index (FMI) with bioelectrical impedance phase angle (PhA). CTF: Magenta, DMF: Green, MPF: Blue, CTF: women without dysmenorrhea, DMF: women with dysmenorrhea, MPF: postmenopausal women.

Regarding the agreement between classic and specific BIVA with DXA body composition parameters, classic BIVA (R/H and Z/H) demonstrated better agreement (Table 3, Figure S1); that is, a negative correlation with FFM_DXA_ (both *r* = −0.834, *p* < 0.01) or FFMI_DXA_ (*r* = −0.863 and *r* = −0.862, *p* < 0.01) in all women, as an increase in the FFM or FFMI causes a decrease in R or Z values. Additionally, specific BIVA (Rsp and Zsp) showed true agreement with fat% (*r* = 0.811 and *r* = 0.810, respectively, *p* < 0.01), FM (both *r* = 0.835, *p* < 0.01), and FMI (*r* = 0.809 and *r* = 0.808, respectively, *p* < 0.01); thus, a positive correlation was observed in all three groups of women (Table 3, Figure S1), as an increase in the body fat causes an increase in the R or Z values. Zonoid depth analysis also demonstrated similar agreement between the DXA measurements and classic or specific BIVA measurements (Figure 1). The menopausal group exhibited significantly lower PhA values than the control and dysmenorrhea groups (Table 1, Figure S2). Moreover, in the control group, PhA positively correlated with certain body composition parameters (Table 3, Figure 2), such as body mass, *r* = 0.772; BMI, *r* = 0.823; FM, *r* = 0.656; FMI, *r* = 0.648; FFM, *r* = 0.734; and FFMI, *r* = 0.905 (in all cases, *p* < 0.05). However, in the dysmenorrhea and postmenopausal groups, PhA did not significantly correlate with any of the body composition parameters.

**TABLE 3:** Correlation between classic, specific BIVA_BIA_, and PhA with body composition.

	Body mass	BMI	Fat%	FM	FMI	FFM	FFMI
All (*n* = 45)							
R/H	**−0.740** *	**−0.672** *	−0.120	**−0.422** *	**−0.370** *	**−0.834** *	**−0.863** *
Xc/H	**−0.421** *	**−0.302** *	−0.117	−0.271	−0.209	**−0.441** *	**−0.333** *
Z/H	**−0.740** *	**−0.672** *	−0.121	−0.422*	**−0.370** *	**−0.834** *	**−0.862** *
Rsp	**0.655** *	**0.677** *	**0.811** *	**0.835** *	**0.809** *	0.247	**0.311** *
Xcsp	**0.629** *	**0.706** *	**0.548** *	**0.657** *	**0.659** *	**0.392** *	**0.560** *
Zsp	**0.654** *	**0.676** *	**0.810** *	**0.835** *	**0.808** *	0.246	**0.310** *
PhA	0.256	**0.315** *	−0.024	0.104	0.121	**0.332** *	**0.472** *
Control (*n* = 13)							
R/H	**−0.771** *	**−0.735** *	−0.305	**−0.596** *	−0.550	**−0.784** *	**−0.838** *
Xc/H	−0.025	0.090	0.175	0.072	0.122	−0.103	0.046
Z/H	**−0.769** *	**−0.732** *	−0.303	**−0.593** *	−0.548	**−0.783** *	**−0.835** *
Rsp	**0.570** *	**0.700** *	**0.834** *	**0.771** *	**0.797** *	0.297	0.518
Xcsp	**0.724** *	**0.839** *	**0.652** *	**0.755** *	**0.774** *	**0.569** *	**0.807** *
Zsp	**0.580** *	**0.712** *	**0.838** *	**0.779** *	**0.806** *	0.306	0.532
PhA	**0.772** *	**0.823** *	0.431	**0.656** *	**0.648** *	**0.734** *	**0.905** *
Dysmenorrhea (*n* = 14)							
R/H	**−0.738** *	**−0.676** *	−0.160	−0.443	−0.389	**−0.794** *	**−0.867** *
Xc/H	−0.519	−0.520	−0.286	−0.409	−0.393	−0.455	**−0.541** *
Z/H	**−0.739** *	**−0.678** *	−0.162	−0.445	−0.391	**−0.795** *	**−0.868** *
Rsp	**0.668** *	**0.776** *	**0.914** *	**0.896** *	**0.902** *	0.193	0.383
Xcsp	**0.654** *	**0.699** *	**0.633** *	**0.721** *	**0.702** *	0.355	0.493
Zsp	**0.660** *	**0.774** *	**0.921** *	**0.897** *	**0.905** *	0.178	0.374
PhA	0.136	0.060	−0.204	−0.049	−0.089	0.286	0.244
Postmenopausal (*n* = 18)							
R/H	**−0.590** *	**−0.581** *	0.058	−0.236	−0.207	**−0.727** *	**−0.857** *
Xc/H	**−0.499** *	−0.401	0.001	−0.223	−0.164	**−0.590** *	**−0.563** *
Z/H	**−0.590** *	**−0.581** *	0.058	−0.236	−0.207	**−0.728** *	**−0.857** *
Rsp	**0.700** *	**0.566** *	**0.705** *	**0.808** *	**0.705** *	0.301	0.161
Xcsp	**0.690** *	**0.635** *	**0.523** *	**0.687** *	**0.620** *	0.415	0.410
Zsp	**0.703** *	**0.560** *	**0.699** *	**0.806** *	**0.698** *	0.309	0.160
PhA	0.111	0.233	−0.085	0.006	0.050	0.178	0.387

*Note*: Values are shown as correlation coefficients. Pearson's or partial correlation (for all participants; *n* = 45) coefficients after adjusting for age were conducted.

Abbreviations: FFM, fat‐free mass; FFMI, fat‐free mass index; FM, fat mass; FMI, fat mass index; R/H and Xc/H, Classic BIVA; R, resistance; Rsp and Xcsp, specific BIVA; Xc, capacitance; Z, impedance vector.

Bold values indicate statistically significant.

* *p* < 0.05.

**FIGURE 3:**
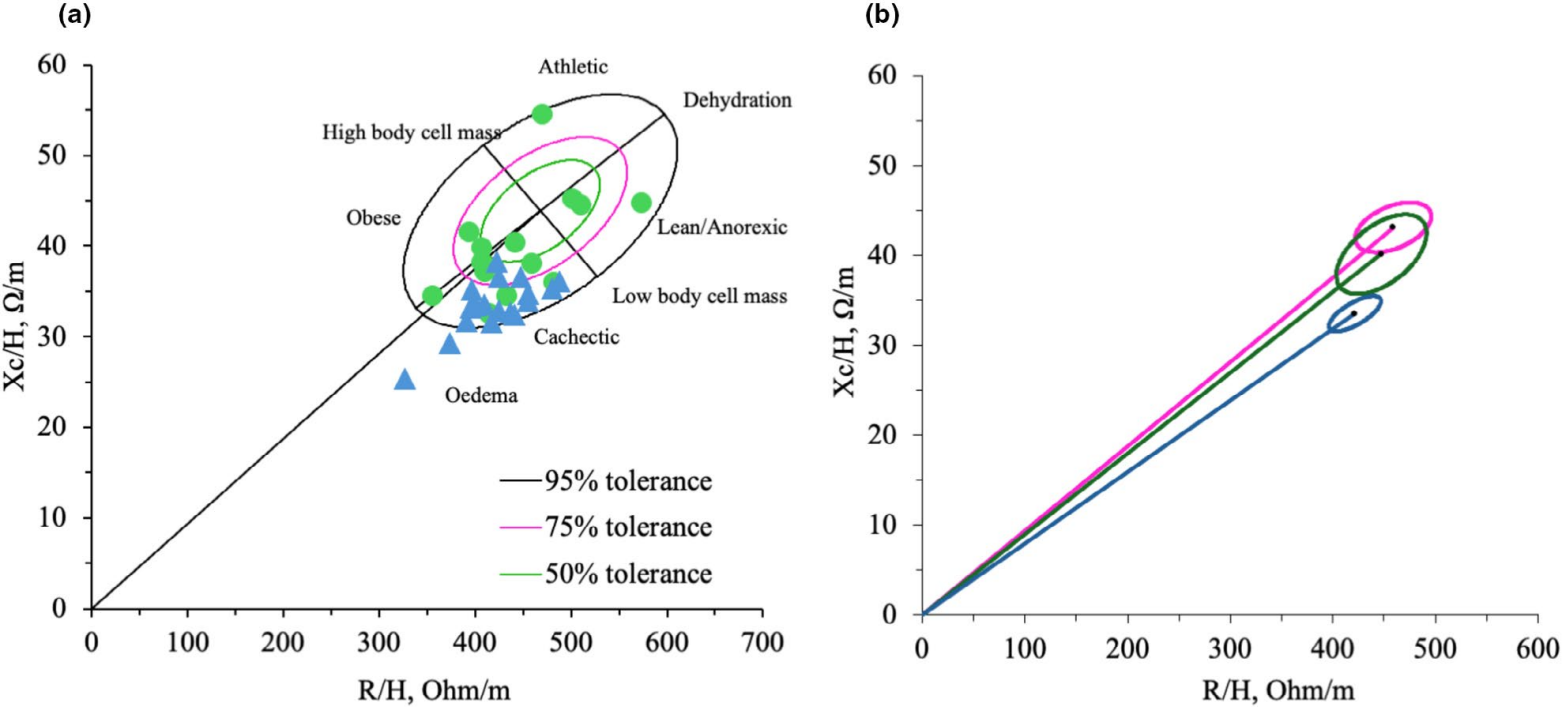
(a) Distribution of impedance vector in the dysmenorrhea and menopausal groups compared to the control group (RXc point graph). CTF: Magenta, DMF: Green, MPF: Blue. CTF: Women without dysmenorrhea, DMF: women with dysmenorrhea, MPF: postmenopausal women. (b) Distribution of the RXc mean graph (95% confidence ellipses) among the three groups of women. CTF: Magenta, DMF: Green, MPF: Blue. CTF: women without dysmenorrhea, DMF: women with dysmenorrhea, MPF: postmenopausal women.

The RXc point graph (Figure 3a) and RXc mean graph (Figure 3b) for the three groups of women were plotted. In the RXc point graph, 16 of the 18 women (88%) in the menopausal group were present in the lower right quadrant, and the remaining participants were outside (lower right quadrant) the 95% tolerance ellipse. In the dysmenorrhea group, 6 of 14 women (42.9%) were in the lower right quadrant, 3 of 14 women (21.4%) were in the upper right quadrant, 4 of 14 women (28.6%) were in the lower left quadrant, and 1 of 14 women (7.1%) was in the upper left corner.

## 4 DISCUSSION

Three different categories of women based on reproductive status were included in this study, which aimed to explore the possible relationships between body composition, bone health, and PhA. The lack of difference between control and dysmenorrhea (Table 1) may be attributed to similar hormonal fluctuations and the limited duration of the study, which may not have allowed the complete manifestation of significant differences. BMD and BMC parameters in the control and dysmenorrhea groups positively correlated with several body composition parameters, but the menopausal group exhibited a negative correlation between fat% and certain BMD and BMC parameters (Table 2). PhA was positively correlated with body composition only in the control group (Table 3, Figure 2). Furthermore, regarding the agreement between classic and specific BIVA with DXA measurements, classic BIVA showed high agreement with the FFM and FFMI, whereas specific BIVA showed good agreement with the body fat parameters measured using DXA (Table 3, Figure 1, and Figure S1). Additionally, in the RXc point graph, all women in the menopausal group fell into the lower right quadrant, indicating edema or lower body cell mass (Figure 3a). Edema, also known as oedema, is a medical term for the accumulation of fluid in the interstitial space of tissue, causing swelling of the body (Stachenfeld, 2014). Postmenopausal women are more prone to edema due to a decrease in estrogen and progesterone levels. Reducing estrogen promotes fluid retention by lowering vasopressin‐mediated osmotic threshold and urine output, while lower progesterone allows aldosterone‐driven water retention (Stachenfeld, 2014). Although reproductive status was self‐reported and not verified by hormonal biomarker, this study provides preliminary evidence that bioelectrical impedance variables may hold promise in identifying body composition during reproductive transition.

### 4.1 Bone health and body composition

Participants in the control group exhibited higher whole‐body BMC or BMD. However, only the menopausal group demonstrated significantly lower BMC or BMD than the control and dysmenorrhea groups (Table 1). Studies have revealed that increased body mass is associated with better bone health (Kerkadi et al., 2022) and serves as a protective factor against osteoporosis and bone fractures (López‐Gómez et al., 2016). Our study indicated that body fat content positively correlated with certain BMD and BMC parameters in the control group, but not in the dysmenorrhea group (Table 2). This weakened relationship between bone health and fat content in women with dysmenorrhea may result from altered dietary patterns. A previous study (Güzeldere et al., 2024) found that inadequate intake of energy, protein, and vitamins, along with excessive consumption of sugary foods, was associated with dysmenorrhea‐like menstrual syndrome and altered BMI in healthy women. The reduced bone health and inverse association with fat content in menopausal women might be due to aging, dietary patterns, and physical activity. A systematic study by Weaver et al. (2016) found that lower consumption of fruits, vegetables, high‐quality proteins, and essential minerals is associated with altered body composition as well as bone health. Adipose tissue plays a crucial role in bone mass development by secreting various hormones such as leptin and adiponectin (López‐Gómez et al., 2016), which stimulate the differentiation of osteoblasts from stem cells in the bone marrow and inhibit the activity of osteoclasts, thereby preventing bone resorption (Rhie et al., 2010). Moreover, Weaver et al. (2016) stated that daily physical activity and adequate sun exposure may substantially reduce the risk of osteoporosis. In this study, classic BIVA showed good agreement with FFM_DXA_ and FFMI_DXA_ but poor agreement with body fat parameters. Specific BIVA showed good agreement with %fat_DXA_, FM_DXA_, and FMI_DXA_ (Table 3, Figure 1, and Figure S2) but poor agreement with FFM_DXA_ and FFMI_DXA_ among these three groups of women. Although these agreements between BIVA and DXA body composition parameters are well known in male and female groups (Buffa et al., 2013) and in older groups (Marini et al., 2013), our study demonstrated this agreement among female groups categorized by reproductive status.

### 4.2 PhA and BIVA

In the present study, the control group demonstrated the highest PhA (5.5 ± 0.5) than the dysmenorrhea (5.1 ± 0.6) and menopausal (4.5 ± 0.3) groups (Table 1). Only the menopausal group showed significantly (*p* < 0.01) lower PhA than the control and dysmenorrhea groups. The lower PhA observed in the menopausal group may be attributed to factors related to aging (Cristina et al., 2005) and a decrease in estrogen and progesterone levels (Geraci et al., 2021). Increasing age is linked to progressive loss of body cell mass and membrane flexibility, which induces electrical resistance and reduces the capacitance property of the cells, resulting in a drop of PhA (Cristina et al., 2005). The decline of estradiol in menopausal women accelerates muscle atrophy by impairing satellite cell proliferation and muscle damage by removing the anti‐inflammatory effect of estrogen (Geraci et al., 2021). The resulting loss of lean tissue diminishes overall tissue capacitance property and lowers the PhA. This study also shows that the dysmenorrhea group had a slightly lower PhA value than the control group, possibly because of the inflammatory action of PGE2 and PGF2α (Gao et al., 2004). PGE2 and PGF2α are responsible for vasoconstriction, tissue hypoxia, and vasodilation, resulting in cellular damage (Ricciotti & Fitzgerald, 2011). In our study, PhA was positively correlated with body composition (Table 3) only in the control group. Bosy‐Wesphal et al. (Anja et al., 2006) reported that an increase in the PhA was associated with an increase in the number of cells. Additionally, healthy cells or muscle mass are responsible for increasing the PhA because of the excellent capacitance of the cells (Khatun et al., 2024).

This is the first study to examine the BIVA graph patterns of female groups based on the reproductive status, that is, the RXc point graph and mean graph. In the RXc point graph (Figure 3a), most participants (88%) in the menopausal group were within the cachectic quadrant or the lower right quadrant, and the remaining participants fell outside the lower right quadrant of the 95% confidence ellipse. The previous study (Cristina et al., 2005) reported that the impedance vectors shift gradually with healthy aging, reflecting a modest decline in lean mass and changes in fluid distribution. However, in our study, 88% of the participants fell out of the 95% confidence ellipse, indicating pathological signals. Research revealed that adequate nutrition (Norman et al., 2012) and resistance training (Fukuda et al., 2016) can improve PhA and shift the impedance vector back toward the healthy reference ellipses in older women. In the dysmenorrhea group, 6 of 14 women (42.9%) were in the lower right quadrant; among them, 4 (28.6%) of 14 participants fell outside the 75% tolerance limit, which refers to overhydration and represents a decrease in body cell mass (Piccoli et al., 1994). The participants who were within the upper right quadrant out of the 95% tolerance limit were recognized as having lower body weight (Piccoli et al., 2005), with a BMI cutoff value of 15 kg/m^2^. In our study, one participant in the dysmenorrhea group fell outside the 75% tolerance limit, and her BMI was 15.7 kg/m^2^. One participant fell in the upper left quadrant within the 95% tolerance limit, indicating adequate hydration and body cell mass (Martins et al., 2021). Additionally, participants who were within the 50% tolerance ellipse represented proper hydration status and healthy body cell mass (Piccoli et al., 2005). In the RXc mean graph (Figure 3b), the menopausal group demonstrated a shorter impedance vector than the control and dysmenorrhea groups. In addition, the ellipses for the menopausal group did not overlap with those of the control and dysmenorrhea groups. As a previous study indicated, a nonoverlapping impedance vector implies a significant difference in the value of PhA (Lukaski, 1996). The impedance vector was slightly higher in the control group than in the dysmenorrhea group; however, the ellipses overlapped.

### 4.3 Limitations

The notable limitation in this study is that the small size of the reference population may introduce bias into the BIVA. To relieve the risk, future studies should include a larger sample size to improve the statistical power and enhance the reliability of the findings. Moreover, self‐reported reproductive status without hormonal confirmation (estrogen, progesterone, and PGs) may limit diagnostic accuracy and their crucial role in regulating body composition and bone health. Additionally, we did not assess the severity of dysmenorrhea symptoms, phases of menstruation, and lifestyle factors such as daily physical activity and dietary pattern, which could have provided deeper insights. The absence of a longitudinal study design limited our ability to observe changes in menstrual health, body composition parameters, and PhA over time. Future research should address these gaps to enhance our understanding of these relationships.

## 5 CONCLUSION

This study offers valuable insights into the impact of reproductive status on body composition, bone health, PhA, and BIVA patterns among the three groups of women. A key finding was the positive relationship between the PhA values and body mass, BMI, FFM, FFMI, and FMI in the control group but not in the dysmenorrhea and menopausal groups. Another important finding was that the R‐Xc point graph demonstrated edema and lower cellular mass in the menopausal group and in approximately 42.9% of the dysmenorrhea group. Thus, it could be inferred that reproductive status was associated with body composition, bone, and cellular health.

## AUTHOR CONTRIBUTIONS

AU, EK, YT, KT, and HS planned the study. SK, AU, EK, YS, SY, YA, DH, NM, KW, KT, and HS collected data. SK, AU, KT, and HS prepared the specimens and analyzed the data. SK and HS conducted the statistical analyses. SK, EK, and HS prepared the illustrations. SK, AU, EK, KT, and HS drafted the manuscript. AMS provided critical comments on the draft. All authors interpreted the results and revised and approved the final version of the manuscript.

## FUNDING INFORMATION

This study was supported by THF grants and the Japan Society for the Promotion of Science KAKENHI for HS (23K27969 and 23KK0177).

## CONFLICT OF INTEREST STATEMENT

Kiyoji Tanaka is the founder and president of THF Co., Ltd., a university‐based start‐up company originating from the University of Tsukuba. THF Co., Inc., financially supported H.S. in conducting this study. The findings of this study are not related to any specific product of the company, and do not endorse any particular product.

## ETHICS STATEMENT

All participants signed a written informed consent form, and the protocol was approved by the Institutional Review Board of the Faculty of Health and Sports Sciences of University of Tsukuba (Ref No Tai 022‐109).

## Supporting information


Figure S1.

Figure S2.

## Data Availability

The datasets generated and/or analyzed in the current study are available from the corresponding author upon reasonable request.
