# Supplementary material for: Exploring the association between bioelectrical impedance parameters and body composition in women with and without dysmenorrhea and postmenopause
Source: Physiol Rep. 2025 Jul 25;13(14):e70473. doi: 10.14814/phy2.70473 (PMC12290943; doi:10.14814/phy2.70473)
Supplement: Supplementary file 1 — Figure S1. [file PHY2-13-e70473-s002.docx]

**Supplementary Figures**

**
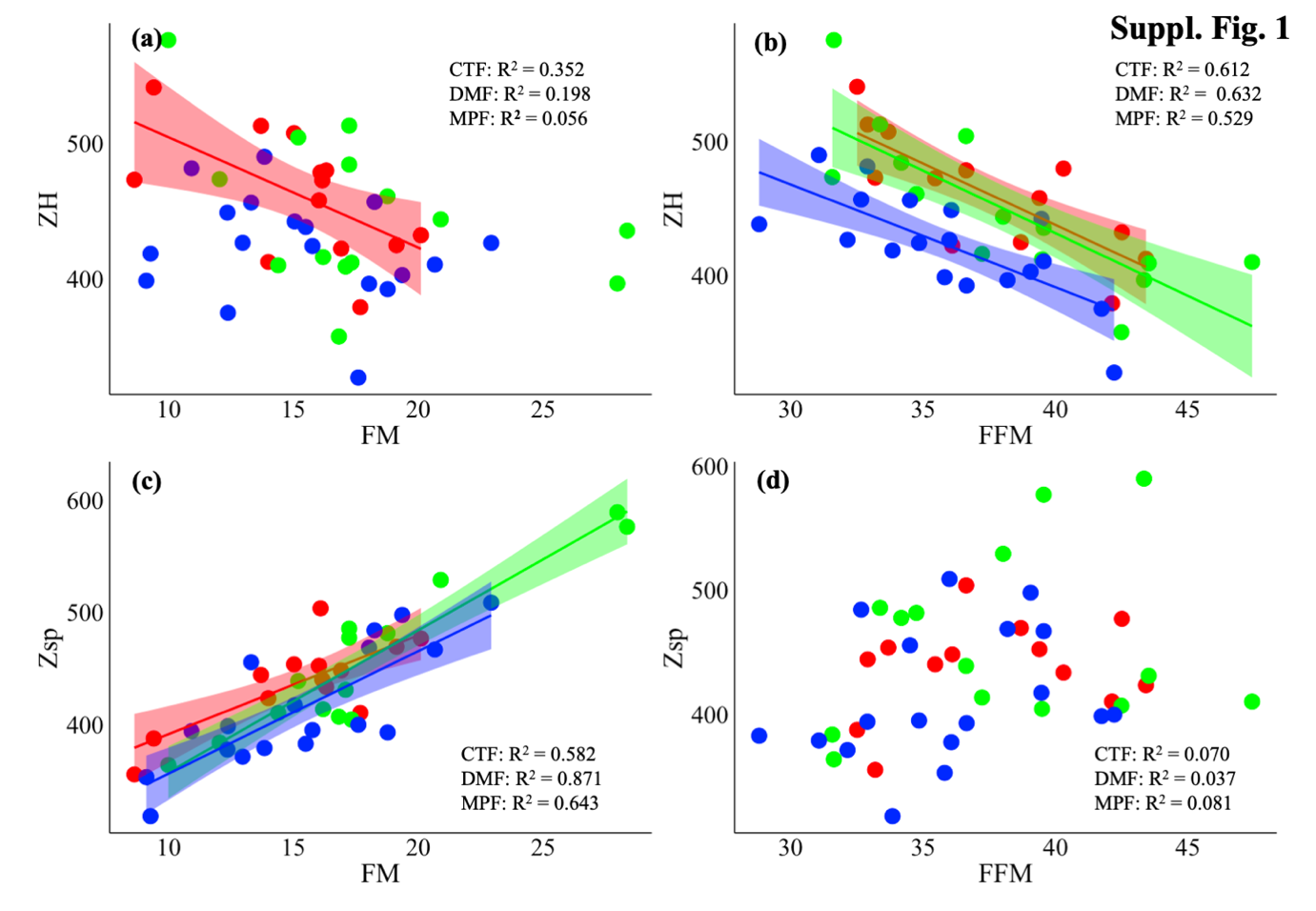
**

**Fig. S1 Correlation between classic (Z/H) or specific (Zsp) impedance vector with Fat mass (FM) and Fat free mass (FFM)**

CTF: Magenta, DMF: Green, MPF: Blue

CTF: women without dysmenorrhea, DMF: women with dysmenorrhea, MPF: post-menopausal women
