# Supplementary material for: Exploring the association between bioelectrical impedance parameters and body composition in women with and without dysmenorrhea and postmenopause
Source: Physiol Rep. 2025 Jul 25;13(14):e70473. doi: 10.14814/phy2.70473 (PMC12290943; doi:10.14814/phy2.70473)
Supplement: Supplementary file 2 — Figure S2. [file PHY2-13-e70473-s001.docx]

**Supplementary Figures**

**
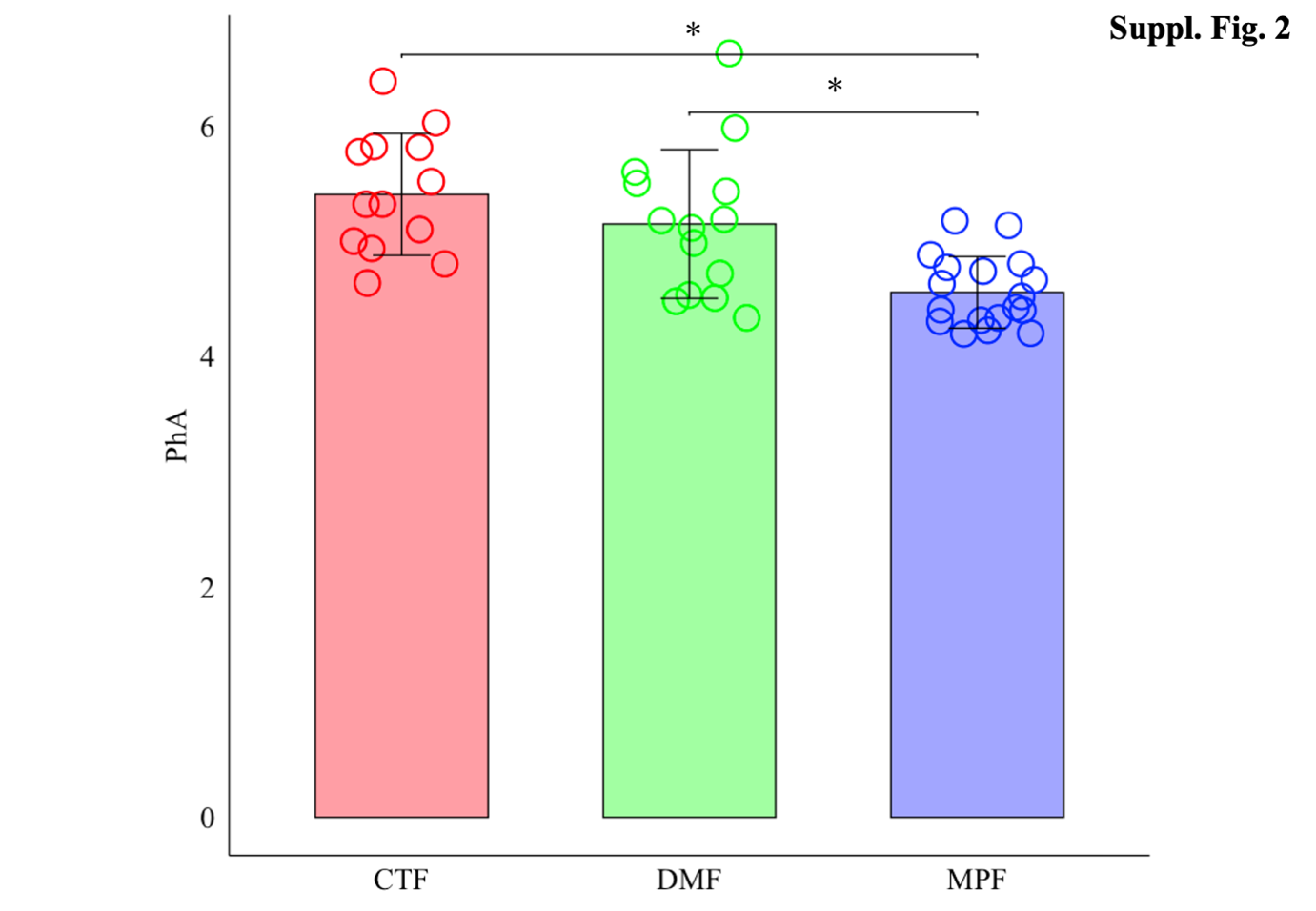
**

**Fig. S2 Value of the phase angle (PhA) among the CTF, DMF, and MPF groups**

CTF: women without dysmenorrhea, DMF: women with dysmenorrhea, MPF: post-menopausal women
